# Trends in resistant Enterobacteriaceae and *Acinetobacter* species in hospitalized patients in the United States: 2013–2017

**DOI:** 10.1186/s12879-019-4387-3

**Published:** 2019-08-23

**Authors:** Vikas Gupta, Gang Ye, Melanie Olesky, Kenneth Lawrence, John Murray, Kalvin Yu

**Affiliations:** 1 Becton, Dickinson and Company, 1 Becton Drive, Franklin Lakes, NJ USA; 2 Tetraphase Pharmaceuticals, Watertown, MA USA

**Keywords:** Antimicrobial resistance, *Acinetobacter*, Enterobacteriaceae, United States, Carbapenems, Extended-spectrum beta-lactamases, Multidrug resistance

## Abstract

**Background:**

Trends in antimicrobial resistance help inform infection control efforts. We examined trends in resistance for Enterobacteriaceae and *Acinetobacter* spp. from 2013 to 2017 in hospitalized US patients.

**Methods:**

We analyzed antimicrobial susceptibility of non-duplicate isolates in hospitalized patients (not limited to hospital-acquired infections) in the US BD Insights Research Database. Resistance profiles of interest were extended-spectrum beta-lactamase (ESBL)-producing, multidrug resistant (MDR), and carbapenem-nonsusceptible (Carb-NS) phenotypes of Enterobacteriaceae, and MDR and Carb-NS *Acinetobacter* spp. Time series models were used to evaluate the patterns of resistance trends in rate per 100 hospital admissions and proportion per isolates tested.

**Results:**

More than 1 million Enterobacteriaceae isolates were obtained from 411 hospitals; 12.05% were ESBL, 1.21% Carb-NS, and 7.08% MDR. Urine was the most common source. For *Acinetobacter* spp. (*n* = 19,325), 37.48% were Carb-NS, 47.66% were MDR, and the most common source was skin/wound cultures. Trend analyses showed that the rates of ESBL and Carb-NS Enterobacteriaceae per 100 hospital admissions increased significantly between 2013 and 2017. Rates of MDR Enterobacteriaceae and Carb-NS and MDR *Acinetobacter* spp. decreased during this time period. Trends in proportions of resistant isolates generally mirrored trends in rates per 100 hospital admissions. MDR Enterobacteriaceae and Carb-NS and MDR *Acinetobacter* spp. were more common in winter than summer.

**Conclusions:**

In this large-scale study of patients in US hospitals, rates of ESBL and Carb-NS Enterobacteriaceae per 100 hospital admissions increased between 2013 and 2017. MDR Enterobacteriaceae and MDR and Carb-NS *Acinetobacter* spp. isolates decreased over this period. These data support continuing infection control and stewardship efforts and the development of new therapeutic options.

**Electronic supplementary material:**

The online version of this article (10.1186/s12879-019-4387-3) contains supplementary material, which is available to authorized users.

## Background

Antibiotic-resistant Gram-negative bacteria have been recognized as a fundamental risk to patient health on both national and global levels [1–3]. Enterobacteriaceae, which account for a significant proportion of infections in hospitalized patients in the US [4, 5], are of particular concern. Carbapenem-resistant Enterobacteriaceae (CRE) have limited treatment options and thus pose a significant clinical dilemma, but extended-spectrum beta-lactamase (ESBL)-producing Enterobacteriaceae and multidrug-resistant (MDR) Enterobacteriaceae are also difficult to treat and occur at a higher prevalence, therefore endangering a greater number of patients [6]. The Centers for Disease Control and Prevention (CDC) categorizes CRE and ESBL-producing Enterobacteriaceae as urgent and serious threats, respectively, and the World Health Organization (WHO) considers them a critical priority for drug development [1–3]. Carbapenem-resistant and MDR *Acinetobacter* are also featured on the CDC and WHO lists of dangerous pathogens [1–3]. Although *Acinetobacter* infections are relatively rare, this pathogen is difficult to treat due to high rates of antibiotic resistance and paucity of options [7].

Trends in resistance patterns provide important insights into emerging pathogens as well as inform public health, infection control, and antimicrobial stewardship approaches. Although comprehensive data on hospital-acquired infections, including catheter-associated urinary tract infections (UTIs) and central-line associated bloodstream infections (BSIs), are available from the US National Healthcare Safety Network (NHSN) [6, 8], national comparative data for antimicrobial-resistant Gram-negative pathogens in the complete population of hospitalized patients are not as easily accessed. The objective of this study was to examine trends in resistance in selected Gram-negative pathogens collected from US hospitals from 2013 through 2017 based on rates of resistance per 100 hospital admissions and proportions of resistant isolates.

## Methods

### Study design and participating hospitals

The study reported here was a retrospective longitudinal analysis of antimicrobial susceptibility of all specified non-duplicate (first isolate of a species within 30 days) Enterobacteriaceae and *Acinetobacter* species (spp.) isolates from hospitalized patients from the first quarter (Q1) of 2013 through the last quarter (Q4) of 2017. Isolates from respiratory, blood, urine, skin/wound, intra-abdominal, and other culture sources were included. Microbiology results likely associated with surveillance cultures (eg, nasal or rectal swabs) and environmental cultures were excluded from this analysis by previously described methodology that uses source, time of collection, pathogen type, and number of pathogens in a culture to flag likely contaminated samples [9]. Reporting institutions consisted of US hospitals in the BD Insights Research Database (Becton, Dickinson and Company, Franklin Lakes, NJ). The clinical research database (formerly referred to as the CareFusion Clinical Research Database) has been described previously [9–11]. This database provides geographical representation across the US; both small and large hospitals in rural and urban areas are included. Susceptibility results and pathogen identification were based on facility reports from hospitals in the database. There was no central laboratory or standardization of breakpoints or testing methods.

Our study evaluated antimicrobial susceptibility in five groups of Gram-negative bacteria using the following definitions as previously described [12, 13]:

1. ESBL Enterobacteriaceae: *Escherichia coli*, *Klebsiella oxytoca*, *Klebsiella pneumoniae*, and *Proteus mirabilis* isolates confirmed as ESBL positive by commercial laboratory panels OR with intermediate susceptibility or resistance to cefepime, ceftazidime, cefotaxime, or ceftriaxone.
2. MDR Enterobacteriaceae: *Citrobacter freundi, Enterobacter aerogenes, Enterobacter cloacae, E. coli, K. oxytoca, K. pneumoniae, Morganella morganii, P. mirabilis,* and *Serratia marcescens* isolates with intermediate susceptibility or resistance to at least one drug in three of the five following classes: aminoglycosides, carbapenems, extended-spectrum cephalosporins, fluoroquinolones, and piperacillin or piperacillin/tazobactam (see Additional file 1 for specific drugs) [6, 14].
3. Carbapenem-nonsusceptible (Carb-NS) Enterobacteriaceae: *C. freundi, E. aerogenes, E. cloacae, E. coli, K. oxytoca, K. pneumoniae, M. morganii, P. mirabilis,* and *S. marcescens* isolates with intermediate susceptibility or resistance to imipenem (excluded for *M. morganii* and *P. mirabilis*), doripenem, ertapenem, or meropenem,. The inclusion of both intermediate susceptibility and resistance is consistent with the CRE definition used by the CDC in their facility guidance document (2012 CRE toolkit), although the 2012 definition did not include ertapenem as a carbapenem [15]. It should be noted that the CDC CRE definition was revised in 2015 to include only Enterobacteriaceae resistant (rather than nonsusceptible) to any carbapenem (doripenem, meropenem, imipenem, or ertapenem) or documented to produce carbapenemase [15].
4. MDR Acinetobacter spp.: *Acinetobacter baumannii/Acinetobacter haemolyticus* (henceforth referred to as *Acinetobacter* spp.) isolates with intermediate susceptibility or resistance to at least one drug in three of the six following classes: ampicillin/sulbactam, aminoglycosides, carbapenems, extended-spectrum cephalosporins, fluoroquinolones, and piperacillin or piperacillin/tazobactam (see Additional file 1).
5. Carb-NS Acinetobacter spp.: *A. baumannii/A. haemolyticus* isolates with intermediate susceptibility or resistance to imipenem, doripenem, or meropenem.

### Outcomes

The outcomes assessed were the rate of bacterial resistance or non-susceptibility, as defined above, per 100 hospital admissions and the proportion of resistant isolates (number of resistant isolates divided by number of non-duplicate isolates tested) for each year-quarter from Q1 2013 through Q4 2017. Hospital admissions, which are frequently used as a denominator in reports of antimicrobial resistance [16, 17], were identified using available census, admission, discharge and transfer files, which were provided by each institution on a more than daily basis.

### Statistical analysis

We employed a two-phase approach to analyze the data. As an exploratory phase, we performed a number of regression modeling analyses with hospital bed size, teaching or non-teaching status, urban or rural status, and geographic locations (regions) as covariates. For the proportion of resistant isolates, we used both logistic regression models and generalized estimating equations (GEE) to estimate resistance. To estimate the resistance rate per 100 admissions, we used a general linear mixed model (GLMM) via Poisson regression model with hospital as random effect. These regression models have the ability to assess effects as well as account for cluster-correlation of data. However, our observed data had a significant autocorrelation (verified via Durbin-Watson test), differencing-autocorrelation, and seasonal or cyclic changing patterns. Although GEE and GLMM can account for within-cluster correlation to some degree (depending on appropriate choice of variance-covariance structures), these methods lack the ability to handle differencing-autocorrelation, seasonality, and periodicity. Therefore, in our second phase of analysis, we decided to use a time series analysis method to fit data. Time series modeling or smoothing methods employ more recent data information (compared with older data) in prediction and estimation of parameters and variances.

In the time series analysis phase, we found that our series data were not stationary or differencing-stationary and thus decided not to use the more popular autoregressive integrated moving average (ARIMA) models. We instead chose the unobserved component model (UCM) [18] to estimate trends in resistance data because our time series data showed certain non-Gaussian characteristics, structural breaks, and outliers. Time series models based on unobserved components are more flexible than regular time series models such as exponential smoothing and ARIMA models, and more effective in handling complex data [19]. Since more hospitals “entered” (contributed data to) the study over time, we postulated that the increasing number of hospitals could affect the estimates of outcome measures. Therefore, in each UCM, we created a time-varying variable, the quarterly number of hospitals contributing data, and modeled this variable as a random effect to see if it was a significant factor of the outcome measures. Our models showed that this time-varying variable had no significant impact.

All model results presented in this paper were generated using the UCM method. All statistical analyses were conducted using Statistical Analysis System (SAS) V9.4 (SAS Institute, Cary, NC) and SAS/ ETS 13.1. *P* values < 0.05 were considered statistically significant.

## Results

A total of 411 hospitals provided data for this study (Table 1). About three-quarters of hospitals (76.6%) were classified as urban, 70.6% were non-teaching hospitals, and the most common bed size was 100 to 300 (42.8%). Geographically, the largest concentration of hospitals was in US Department of Health & Human Services Region 4 (south central states; 23.6%) and Region 5 (north central states; 23.4%) (Table 1).

**Table 1:** Distribution of Hospitals Included in the Study

Characteristic	n	%
Overall	411	100
HHS region (states)^a^		
Region 1 (CT, ME, MA, NH, RI, VT)	9	2.2
Region 2 (NJ, NY)	45	11.0
Region 3 (DE, DC, MD, PA, VA, WV)	18	4.4
Region 4 (AL, FL, GA, KY, MS, NC, SC, TN)	97	23.6
Region 5 (IL, IN, MI, MN, OH, WI)	96	23.4
Region 6 (AR, LA, NM, OK, TX)	70	17.0
Region 7 (IA, KS, MO, NE)	12	2.9
Region 8 (CO, MT, ND, SD, UT, WY)	12	2.9
Region 9 (AZ, CA, HI, NV)	34	8.3
Region 10 (AK, ID, OR, WA)	18	4.4
Urban/Rural		
Urban	315	76.6
Rural	96	23.4
Teaching status		
Non-teaching	290	70.6
Teaching	121	29.4
Bed size		
< 100	109	26.5
100–300	176	42.8
> 300	126	30.7

Abbreviations: *HHS* US Department of Health & Human Services

^a^ US Territories were not included

More than 1 million Enterobacteriaceae isolates were evaluated for ESBL (1,112,312 tested), Carb-NS (1,275,311 tested), and MDR (1,275,311 tested). Over the 5-year period, 12.05% Enterobacteriaceae isolates were identified as ESBL phenotype (hereafter referred to as ESBL Enterobacteriaceae), 1.21% as Carb-NS, and 7.08% as MDR (Table 2). A total of 19,325 *Acinetobacter* spp. isolates were tested for Carb-NS and MDR. Of these isolates, 37.48% isolates were identified as Carb-NS and 47.66% as MDR.

**Table 2:** Distribution of Pathogens by Culture Source

Source	Enterobacteriaceae						*Acinetobacter* spp.			
	ESBL		Carb-NS		MDR		Carb-NS		MDR	
	n (N Tested)	%	n (N Tested)	%	n (N Tested)	%	n (N Tested)	%	n (N Tested)	%
All sources	134,032 (1,112,312)	12.05%	15,460 (1,275,311)	1.21%	90,327 (1,275,311)	7.08%	7243 (19,325)	37.48%	9210 (19,325)	47.66%
Urine	89,224 (775,699)	11.50%	7092 (843,193)	0.84%	56,944 (843,193)	6.75%	984 (3201)	30.74%	1352 (3201)	42.24%
Skin/wound	15,937 (122,271)	13.03%	3174 (167,125)	1.90%	11,852 (167,125)	7.09%	2613 (7024)	37.20%	3319 (7024)	47.25%
Respiratory	10,325 (59,316)	17.41%	3015 (87,605)	3.44%	8959 (87,605)	10.23%	2893 (6429)	45.00%	3619 (6429)	56.29%
Blood	13,654 (105,998)	12.88%	1368 (119,329)	1.15%	9167 (119,329)	7.68%	532 (1968)	27.03%	660 (1968)	33.54%
Other sources	2895 (24,343)	11.89%	450 (29,505)	1.53%	2042 (29,505)	6.92%	143 (504)	28.37%	172 (504)	34.13%
Intra-abdominal	1997 (24,685)	8.09%	361 (28,554)	1.26%	1363 (28,554)	4.77%	78 (199)	39.20%	88 (199)	44.22%

Abbreviations: *Carb-NS* carbapenem-nonsusceptible, *ESBL* extended-spectrum beta-lactamase-producing, *MDR* multidrug resistant

For Enterobacteriaceae, urine cultures accounted for the majority of resistant isolates (66.66% ESBL, 45.87% Carb-NS, and 63.04% MDR), followed by skin/wound (11.89, 20.53, and 13.12%, respectively) (Table 2). The highest rates of resistance were observed in respiratory cultures (17.41, 3.44, and 10.23%, and for ESBL, Carb-NS, and MDR, respectively). For *Acinetobacter* spp., respiratory cultures were the most common source of Carb-NS (39.94%) and MDR (39.29%), and also had the highest proportion of Carb-NS and MDR isolates (45.00 and 56.29%, respectively) (Table 2). Skin/wound was the second most common source of resistant *Acinetobacter* spp. (36.08% for Carb-NS, 36.04% for MDR).

Observational data for rates and proportions of antibiotic-resistant Enterobacteriaceae and *Acinetobacter* spp. from 2013 to 2017 are shown by quarter in Additional files 2 and 3, respectively.

### Trends in antibiotic-resistant Enterobacteriaceae

**Fig. 1:**
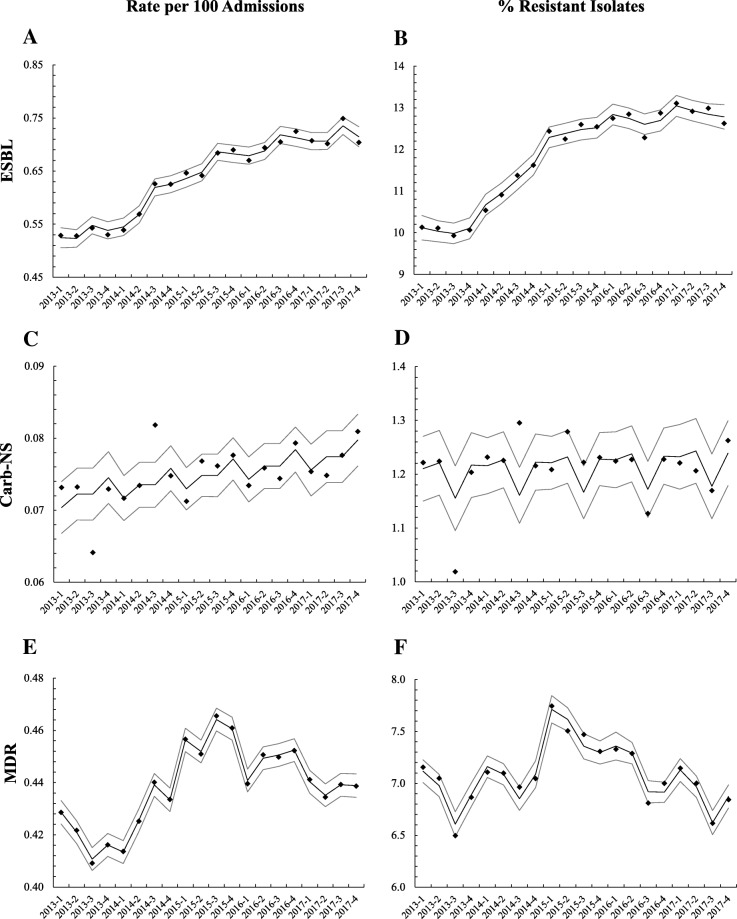
Observed and model-estimated resistance trends in antibiotic-resistant Enterobacteriaceae by year-quarter. **a** Extended-spectrum beta-lactamase (ESBL)-producing Enterobacteriaceae per 100 hospital admissions and (**b**) as a proportion of tested isolates; (**c**) carbapenem-nonsusceptible (Carb-NS) Enterobacteriaceae per 100 hospital admissions and (**d**) as a proportion of tested isolates; and (**e**) multidrug-resistant (MDR) Enterobacteriaceae per 100 hospital admissions and (**f**) as a proportion of tested isolates; and. Diamonds indicate observed data, solid lines indicate predicted trends, and dotted lines indicate 95% confidence intervals

Between 2013 and 2017, the rate of ESBL per 100 hospital admissions increased significantly in US hospitals. Statistical model-based assessment showed that the overall (linear) trend increased over time with an average slope of 0.0089/quarter (*p* < 0.0001); no significant seasonal pattern was observed (Table 3 and Fig. 1a). Evaluations of the proportions of ESBL Enterobacteriaceae isolates (number of resistant isolates divided by number of isolates tested) found that ESBL Enterobacteriaceae increased from 10.1% Q1 2013 to 12.6% in Q4 2017 (Additional file 2), with a similar pattern to that observed for rate per 100 hospital admissions (average slope of 0.151%/quarter; *p* < 0.0001) (Table 3 and Fig. 1b). Seasonal variations were not significant.

**Table 3:** Model-detected Trends in Patterns of Resistance in Enterobacteriaceae and *Acinetobacter* spp. from 2013 to 2017

Pathogen Class	Pathogen	Measurement	Overall (5-year) linear trend			Seasonality	
			Pattern	Quarterly slope (95% CI)	*p*	Pattern	*p*
Enterobacteriaceae	ESBL	Rate per 100 admissions	Increasing	0.0089 (0.0052, 0.0127)	< 0.0001	Insignificant	0.0617
		% of isolates tested	Increasing	0.1510 (0.0974, 0.2045)	< 0.0001	Insignificant	0.1168
	Carb-NS	Rate per 100 admissions	Increasing	0.0004 (0.0001, 0.0006)	0.0047	Insignificant	0.5075
		% of isolates tested	Insignificant	0.0013 (−0.0027, 0.0052)	0.5331	Insignificant	0.1301
	MDR	Rate per 100 admissions	Decreasing	−0.0022 (−.0038, −.0006)	0.0066	Insignificant	0.0501
		% of isolates tested	Decreasing	−0.0273 (− 0.0499, − 0.0048)	0.0176	Q1 higher, Q3 lower	0.0010
*Acinetobacter* spp.	Carb-NS	Rate per 100 admissions	Decreasing	−0.0009 (− 0.0011, − 0.0006)	< 0.0001	Insignificant	0.1421
		% of isolates tested	Insignificant	−0.1075 (− 0.0904, 0.0697)	0.2345	Q1 higher, Q3 lower	< 0.0001
	MDR	Rate per 100 admissions	Decreasing	−0.0014 (− 0.0017, − 0.0010)	< 0.0001	Insignificant	0.2189
		% of isolates tested	Decreasing	−0.4269 (− 0.5793, − 0.2744)	< 0.0001	Q1 higher, Q3 lower	< 0.0001

Abbreviations: *Carb-NS* carbapenem-nonsusceptible, *CI* confidence interval, *ESBL* extended-spectrum beta-lactamase-producing, *MDR* multidrug resistant, *Q* quarter

Carb-NS Enterobacteriaceae also showed significant increases in rate per 100 hospital admissions between 2013 and 2017, although the trend was more gradual than observed with ESBL pathogens (slope of 0.0004/quarter; *p* = 0.0047) (Table 3 and Fig. 1c). Increases in the proportion of Carb-NS Enterobacteriaceae were not significant (1.2% Q1 2013 to 1.3% Q4 2017, *p* = 0.5331) (Table 3 and Fig. 1d). No significant seasonal variations were observed for either rate or proportion of resistance in Carb-NS Enterobacteriaceae.

The rate of MDR Enterobacteriaceae per 100 hospital admissions showed an “up-down” nonlinear changing pattern, increasing prior to 2015 and decreasing during the most recent 3-year time period (Table 3 and Fig. 1e). The overall trend between 2013 and 2017 was a slight but significant decrease in both rates per 100 admissions (slope of − 0.0022; *p* = 0.0066) and proportion of resistant pathogens (slope of − 0.0273; *p* = 0.0176) (Table 3 and Fig. 1e and f). The proportion of resistant isolates showed modest but significant seasonal variation, with higher rates in the winter period (Q1) compared with the summer period (Q3) (Table 3).

### Trends in antibiotic-resistant *Acinetobacter* spp.

**Fig. 2:**
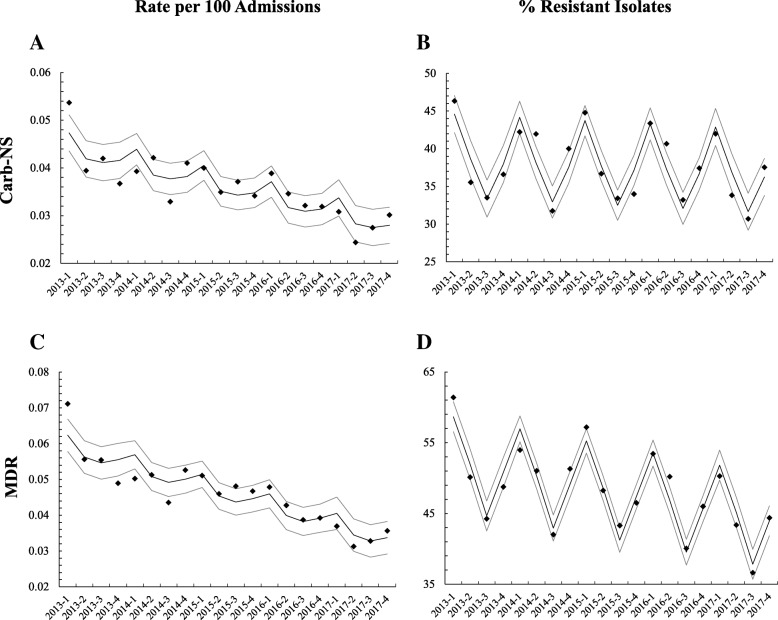
Observed and model-estimated resistance trends in *Acinetobacter* spp. by year-quarter. **a** Carbapenem-nonsusceptible (Carb-NS) *Acinetobacter* spp. per 100 hospital admissions and (**b**) as a proportion of tested isolates; (**c**) multidrug-resistant (MDR) *Acinetobacter* spp. per 100 hospital admissions and (**d**) as a proportion of tested isolates;. Diamonds indicate observed data, solid lines indicate predicted trends, and dotted lines indicate 95% confidence intervals

Both Carb-NS and MDR *Acinetobacter* spp. showed an overall significant linear decreasing trend in rates per 100 hospital admissions from 2013 to 2017 (Table 3, Fig. 2), with slopes of − 0.0009/quarter and − 0.00134/ quarter, respectively (both *p* < 0.0001) (Fig. 2a and c). The proportions of Carb-NS *Acinetobacter* spp. also showed an overall decreasing pattern, but the trend was insignificant (*p* = 0.2345) (Table 3 and Fig. 2b). Decreases in the proportions of MDR *Acinetobacter* spp. did achieve significance (*p* < 0.0001) (Table 3 and Fig. 2d). Both Carb-NS and MDR *Acinetobacter* spp. showed a strong seasonal changing pattern in the proportion of resistant isolates in which higher resistance rates were observed in the winter period (Q1) compared with the summer season (Q3) (Table 3 and Fig. 2b and d).

## Discussion

Antimicrobial resistance in Gram-negative pathogens continues to threaten public health and increase the societal cost of health care. Carbapenem-resistant pathogens are a particular problem due to limited treatment options, ability to infect multiple organ systems, and high attributable mortality and cost [13, 20]. Although CRE are unquestionably an important clinical concern, ESBL and MDR Enterobacteriaceae pose a substantial threat to patient health and are present in greater numbers [1, 6]. Based on our Q4 2017 data, these pathogens accounted for a 10-fold higher incidence of pathogens in hospitalized patients than Carb-NS Enterobacteriaceae. *Acinetobacter* spp., a rare but dangerous Gram-negative pathogen, is associated with high rates of resistance and mortality [7, 21].

Information on trends in susceptibility is important from public health, infection prevention, and antimicrobial development standpoints. Some recent reports suggest that rates of antimicrobial-resistant Enterobacteriaceae are decreasing, including an NHSN study of central line-associated BSIs and catheter-associate UTIs [8]. However, other studies suggest a continued increase in the rate of antimicrobial-resistant Gram-negative pathogens [22–24]. Outside of hospitals, there has been an increase in antibiotic-resistant Gram-negative pathogens in poultry and livestock [25], which may contribute to the spread of infections in the community.

Our study evaluated the rates of antimicrobial-resistant or nonsusceptible Enterobacteriaceae and *Acinetobacter* spp. from multiple culture sources in hospitalized patients throughout the United States from 2013 to 2017. We found that rates of ESBL and Carb-NS Enterobacteriaceae per 100 hospital admissions increased significantly during this time period, although increases were fairly modest, while rates of MDR Enterobacteriaceae, MDR *Acinetobacter* spp., and Carb-NS *Acinetobacter* spp. decreased. Analyses of the proportions of resistant isolates largely mirrored the trends observed with rates per 100 hospital admission, although increases in the proportion of Carb-NS Enterobacteriaceae and decreases in the proportion of Carb-NS *Acinetobacter* spp. did not reach statistical significance. For *Acinetobacter* spp., decreases were fairly constant throughout the 5-year period. In contrast, MDR Enterobacteriaceae rates and proportion of resistant isolates initially increased followed by a decrease from Q2 2015 to Q4 2017. Although this recent trend is encouraging, decreases in MDR Enterobacteriaceae were modest and rates are still high (6.8% in Q4 2017).

Independent reports of antimicrobial resistance and non-susceptibility are difficult to compare because they are influenced by a number of factors, including number of participating institutions, culture site(s), pathogens evaluated, definitions of resistance, culture ordering practices, and pathogen sources (community- versus hospital-acquired or surveillance versus infection). There are thus several reasons why our findings of increases in ESBL and Carb-NS Enterobacteriaceae might differ from studies reporting decreased rates of antimicrobial-resistant Enterobacteriaceae. Most notable is that our analyses include cultures collected in the admission period as well as those cultured in the hospital-onset time period; our analyses were confined to hospitalized patients, but were not specific to hospital-acquired infections. In contrast, CDC reports of hospital-acquired infections, by definition, attempt to exclude patients admitted with infections. In many facilities, hospital-acquired infections are targeted for intensive infection control and antimicrobial stewardship efforts, which may result in decreased antibiotic resistance in these infections compared with the resistance profile of pathogens in the community at large. The geographic representation of included hospitals can also influence results. We have shown previously that there are significant differences in rates of antibiotic-resistant *Acinetobacter* spp. and MDR Enterobacteriaceae across geographic regions in the US [12].

Our results concerning recent decreases in antibiotic-resistant *Acinetobacter* infections are consistent with other observations [6, 26]. Others have also observed seasonal variations in US *Acinetobacter* infections [27, 28], but previous studies have reported increased infection rates in summer months compared with winter months, whereas we found increased proportions of resistant pathogens (but not rates per 100 admissions) in the winter months. We observed a similar pattern for MDR Enterobacteriaceae, consistent with a report on antibiotic-resistant *E. coli* in which higher proportions of resistant isolates in winter months correlated with antibiotic prescribing practices [29]. The reasons for discrepancy in seasonal trends among different studies may involve differences in study methodology, in particular the inclusion of admission period cultures rather than solely “hospital onset” isolates and assessment of the proportion of resistant isolates versus overall frequencies of infection.

Limitations of our study include the collection and analysis of data from non-duplicated unique collected cultures rather than from unique patients. We are therefore unable to evaluate clinical outcomes associated with the antibiotic-resistant pathogens in this study. The results reported here represent non-duplicate culture positive isolates and not confirmed invasive infections. Our study included only selected *Acinetobacter* spp., and mechanisms of resistance were not investigated. Susceptibility was based on local microbiology practices at each facility and not standardized across facilities. Enterobacteriaceae ESBL and CRE testing practices and breakpoints are known to vary among different institutions [30], and institutions with lower breakpoints typically report higher resistance rates [31]. In particular, delayed implementation of updated Clinical & Laboratory Standards Institute guidelines for third-generation cephalosporins, cefepime, and carbapenem breakpoints may have influenced susceptibility assessments. These breakpoints were lowered in 2010 (carbapenems and third-generation cephalosporins) and 2014 (cefepime) for Enterbacteriaceae and in 2014 (carbapenems) for *Acinetobacter* spp., but immediate adoption in hospital laboratories likely varied due to the use of automated antimicrobial susceptibility testing systems. In one study of 25 US community hospitals, only 5 (20%) had adopted the 2010 carbapenem breakpoints by the end of 2012 [31]. It is possible that the increased resistance observed in ESBL and Carb-NS Enterobacteriaceae in our study may reflect the expanding adoption of these lower breakpoints, leading to the identification of a greater number of isolates as antibiotic resistant. The inclusion of isolates with intermediate resistance increased the number of isolates slightly, so our data of nonsusceptible isolates cannot be strictly compared to studies of pathogens meeting criteria for resistance. Antimicrobial susceptibility results for ertapenem may have also influenced Carb-NS rates, as AmpC hyperproduction and porin changes can cause some isolates to be Carb-NS to ertapenem while remaining susceptible to other carbapenems [32]. Despite these limitations, we believe our study provides an epidemiological lens on trends in antimicrobial resistance that reflects both the community and inpatient burden of hospitalized patients and may help inform further studies.

Our findings highlight the multiple factors influencing infections with resistant pathogens. Antibiotic-resistant Gram-negative bacteria are found in multiple culture sources, often spread from non-infected fomites and contacts, and can be ubiquitously represented in intensive care, medical, and surgical hospital wards [33]. Although understandably a focus of attention, reportable healthcare-acquired infections represent only a portion of clinically relevant infections. The inclusion of multiple sources of positive cultures may represent a more accurate risk of spreading drug-resistant organisms between patients, healthcare workers and family members [34, 35] or from community food supplies [25]. Additional Gram-negative bacteria, particularly *Pseudomonas aeruginosa*, also pose important clinical challenges. Work is in progress to explore trends in antibiotic-resistant *P. aeruginosa*.

## Conclusion

Our findings from this large-scale study of patients in US hospitals show increasing numbers of infections due to ESBL and Carb-NS Enterobacteriaceae between 2013 and 2017. These data support continuing efforts by the CDC and WHO to combat these pathogens. Infections caused by MDR and Carb-NS *Acinetobacter* spp. are decreasing, but *Acinetobacter* remains a dangerous and difficult-to-treat pathogen. Continued infection control efforts, together with diagnostic and antimicrobial stewardship and new antibiotics to expand treatment options, will be required to manage these antibiotic-resistant Gram-negative pathogens.

## Additional files

Additional file 1: Specific antibiotics for multidrug resistance. (DOCX 18 kb)

Additional file 2: Descriptive statistics of resistance in Enterobacteriaceae over time. Rate indicates resistance per 100 admissions and % indicates proportion of resistant isolates (resistant isolates/total isolates tested). (DOCX 23 kb)

Additional file 3: Descriptive statistics of resistance in *Acinetobacter* spp. over time. Rate indicates resistance per 100 admissions and % indicates proportion of resistant isolates (resistant isolates/total isolates tested). (DOCX 22 kb)

## Data Availability

The datasets used and/or analysed during the current study are available from the corresponding author on reasonable request.

## Abbreviations

ARIMA: Autoregressive integrated moving average
BD: Becton, Dickinson and Company
BSI: Bloodstream infection
Carb-NS: Carbapenem-nonsusceptible
CDC: Centers for Disease Control and Prevention
CRE: Carbapenem-resistant Enterobacteriaceae
ESBL: Extended-spectrum beta-lactamase
GEE: Generalized estimating equation
GLMM: General linear mixed model
MDR: Multidrug resistant
NHSN: US National Healthcare Safety Network
Q: Quarter
SAS: Statistical Analysis System
UCM: Unobserved component model
UTI: Urinary tract infection
WHO: World Health Organization
