# Supplementary material for: Trends in resistant Enterobacteriaceae and Acinetobacter species in hospitalized patients in the United States: 2013–2017
Source: BMC Infect Dis. 2019 Aug 23;19:742. doi: 10.1186/s12879-019-4387-3 (PMC6708167; doi:10.1186/s12879-019-4387-3)
Supplement: Supplementary file 1 — Specific antibiotics for multidrug resistance. (DOCX 18 kb) [file 12879_2019_4387_MOESM1_ESM.docx]

**Additional file 1.** Specific antibiotics for multidrug resistance.

| **Drug class** | **MDR Enterobacteriaceae** | **MDR *Acinetobacter* spp** |
| --- | --- | --- |
| Extended-spectrum cephalosporins | Cefepime  Cefotaxime  Ceftazidime  Ceftriaxone | Ceftazidime  Cefepime |
| Fluoroquinolones | Ciprofloxacin  Levofloxacin  Moxifloxacin | Ciprofloxacin  Levofloxacin |
| Aminoglycosides | Amikacin  Gentamicin  Tobramycin | Amikacin  Gentamicin  Tobramycin |
| Carbapenems | Ertapenem  Imipenem  Meropenem  Doripenem | Imipenem  Meropenem  Doripenem |

Abbreviations: MDR, multidrug resistant
