# Supplementary material for: Trends in resistant Enterobacteriaceae and Acinetobacter species in hospitalized patients in the United States: 2013–2017
Source: BMC Infect Dis. 2019 Aug 23;19:742. doi: 10.1186/s12879-019-4387-3 (PMC6708167; doi:10.1186/s12879-019-4387-3)
Supplement: Supplementary file 2 — Descriptive statistics of resistance in Enterobacteriaceae over time. Rate indicates resistance per 100 admissions and % indicates proportion of resistant isolates (resistant isolates/total isolates tested). (DOCX 23 kb) [file 12879_2019_4387_MOESM2_ESM.docx]

**Additional file 2.** Descriptive statistics of resistance in Enterobacteriaceae over time. Rate indicates resistance per 100 admissions and % indicates proportion of resistant isolates (resistant isolates/total isolates tested).

| **Year-Q** | **Number of Hospitals** | **Number of Admissions** | **ESBL** | | | **Carb-NS** | | | **MDR** | | |
| --- | --- | --- | --- | --- | --- | --- | --- | --- | --- | --- | --- |
|  |  |  | **n (N tested)** | **Rate** | **%** | **n (N tested)** | **Rate** | **%** | **n (N tested)** | **Rate** | **%** |
| 2013-1 | 229 | 739,427 | 3,910 (38,584) | 0.529 | 10.1 | 541 (44,290) | 0.073 | 1.2 | 3,169 (44,290) | 0.429 | 7.2 |
| 2013-2 | 237 | 766,179 | 4,046 (40,005) | 0.528 | 10.1 | 561 (45,840) | 0.073 | 1.2 | 3,231 (45,840) | 0.422 | 7.1 |
| 2013-3 | 242 | 802,971 | 4,358 (43,867) | 0.543 | 9.9 | 515 (50,564) | 0.064 | 1.0 | 3,285 (50,564) | 0.409 | 6.5 |
| 2013-4 | 245 | 803,347 | 4,257 (42,283) | 0.530 | 10.1 | 586 (48,698) | 0.073 | 1.2 | 3,343 (48,698) | 0.416 | 6.9 |
| 2014-1 | 253 | 814,703 | 4,390 (41,655) | 0.539 | 10.5 | 584 (47,420) | 0.072 | 1.2 | 3,370 (47,420) | 0.414 | 7.1 |
| 2014-2 | 267 | 866,133 | 4,929 (45,168) | 0.569 | 10.9 | 636 (51,891) | 0.073 | 1.2 | 3,683 (51,891) | 0.425 | 7.1 |
| 2014-3 | 275 | 911,490 | 5,705 (50,128) | 0.626 | 11.4 | 746 (57,593) | 0.082 | 1.3 | 4,011 (57,593) | 0.440 | 7.0 |
| 2014-4 | 289 | 941,362 | 5,885 (50,627) | 0.625 | 11.6 | 704 (57,920) | 0.075 | 1.2 | 4,081 (57,920) | 0.434 | 7.1 |
| 2015-1 | 295 | 965,585 | 6,242 (50,177) | 0.646 | 12.4 | 688 (56,937) | 0.071 | 1.2 | 4,409 (56,937) | 0.457 | 7.7 |
| 2015-2 | 313 | 990,306 | 6,353 (51,847) | 0.642 | 12.3 | 761 (59,514) | 0.077 | 1.3 | 4,466 (59,514) | 0.451 | 7.5 |
| 2015-3 | 321 | 1,058,467 | 7,240 (57,461) | 0.684 | 12.6 | 806 (65,959) | 0.076 | 1.2 | 4,927 (65,959) | 0.466 | 7.5 |
| 2015-4 | 345 | 1,066,363 | 7,356 (58,637) | 0.690 | 12.5 | 828 (67,269) | 0.078 | 1.2 | 4,915 (67,269) | 0.461 | 7.3 |
| 2016-1 | 362 | 1,153,368 | 7,726 (60,606) | 0.670 | 12.8 | 847 (69,186) | 0.073 | 1.2 | 5,070 (69,186) | 0.440 | 7.3 |
| 2016-2 | 369 | 1,155,957 | 8,022 (62,450) | 0.694 | 12.9 | 877 (71,476) | 0.076 | 1.2 | 5,209 (71,476) | 0.451 | 7.3 |
| 2016-3 | 373 | 1,181,199 | 8,325 (67,772) | 0.705 | 12.3 | 879 (78,016) | 0.074 | 1.1 | 5,313 (78,016) | 0.450 | 6.8 |
| 2016-4 | 386 | 1,200,083 | 8,698 (67,559) | 0.725 | 12.9 | 952 (77,537) | 0.079 | 1.2 | 5,427 (77,537) | 0.452 | 7.0 |
| 2017-1 | 390 | 1,263,105 | 8,930 (68,133) | 0.707 | 13.1 | 952 (77,986) | 0.075 | 1.2 | 5,572 (77,986) | 0.441 | 7.1 |
| 2017-2 | 400 | 1,263,981 | 8,867 (68,655) | 0.702 | 12.9 | 946 (78,435) | 0.075 | 1.2 | 5,491 (78,435) | 0.434 | 7.0 |
| 2017-3 | 405 | 1,293,205 | 9,687 (74,583) | 0.749 | 13.0 | 1,004 (85,852) | 0.078 | 1.2 | 5,680 (85,852) | 0.439 | 6.6 |
| 2017-4 | 411 | 1,293,657 | 9,106 (72,115) | 0.704 | 12.6 | 1,047 (82,928) | 0.081 | 1.3 | 5,675 (82,928) | 0.439 | 6.8 |

Abbreviations: Carb-NS, carbapenem-nonsusceptible; ESBL, extended-spectrum beta-lactamase-producing; MDR, multidrug resistant; Q, quarter
