# Supplementary material for: Trends in resistant Enterobacteriaceae and Acinetobacter species in hospitalized patients in the United States: 2013–2017
Source: BMC Infect Dis. 2019 Aug 23;19:742. doi: 10.1186/s12879-019-4387-3 (PMC6708167; doi:10.1186/s12879-019-4387-3)
Supplement: Supplementary file 3 — Descriptive statistics of resistance in Acinetobacter spp. over time. Rate indicates resistance per 100 admissions and % indicates proportion of resistant isolates (resistant isolates/total isolates tested). (DOCX 22 kb) [file 12879_2019_4387_MOESM3_ESM.docx]

**Additional file 3**. Descriptive statistics of resistance in *Acinetobacter* spp. over time. Rate indicates resistance per 100 admissions and % indicates proportion of resistant isolates (resistant isolates/total isolates tested).

| **Year-Q** | **Number of Hospitals** | **Number of Admissions** | **Carb-NS** | | | **MDR** | | |
| --- | --- | --- | --- | --- | --- | --- | --- | --- |
|  |  |  | **n (N tested)** | **Rate** | **%** | **n (N tested)** | **Rate** | **%** |
| 2013-1 | 229 | 739,427 | 397 (857) | 0.054 | 46.3 | 526 (857) | 0.071 | 61.4 |
| 2013-2 | 237 | 766,179 | 302 (850) | 0.039 | 35.5 | 426 (850) | 0.056 | 50.1 |
| 2013-3 | 242 | 802,971 | 337 (1,006) | 0.042 | 33.5 | 445 (1,006) | 0.055 | 44.2 |
| 2013-4 | 245 | 803,347 | 295 (806) | 0.037 | 36.6 | 393 (806) | 0.049 | 48.8 |
| 2014-1 | 253 | 814,703 | 320 (758) | 0.039 | 42.2 | 409 (758) | 0.050 | 54.0 |
| 2014-2 | 267 | 866,133 | 365 (870) | 0.042 | 42.0 | 444 (870) | 0.051 | 51.0 |
| 2014-3 | 275 | 911,490 | 300 (945) | 0.033 | 31.8 | 397 (945) | 0.044 | 42.0 |
| 2014-4 | 289 | 941,362 | 386 (965) | 0.041 | 40.0 | 495 (965) | 0.053 | 51.3 |
| 2015-1 | 295 | 965,585 | 386 (862) | 0.040 | 44.8 | 493 (862) | 0.051 | 57.2 |
| 2015-2 | 313 | 990,306 | 346 (943) | 0.035 | 36.7 | 455 (943) | 0.046 | 48.3 |
| 2015-3 | 321 | 1,058,467 | 393 (1,176) | 0.037 | 33.4 | 509 (1,176) | 0.048 | 43.3 |
| 2015-4 | 345 | 1,066,363 | 364 (1,071) | 0.034 | 34.0 | 498 (1,071) | 0.047 | 46.5 |
| 2016-1 | 362 | 1,153,368 | 448 (1,033) | 0.039 | 43.4 | 552 (1,033) | 0.048 | 53.4 |
| 2016-2 | 369 | 1,155,957 | 400 (984) | 0.035 | 40.7 | 494 (984) | 0.043 | 50.2 |
| 2016-3 | 373 | 1,181,199 | 379 (1,141) | 0.032 | 33.2 | 457 (1,141) | 0.039 | 40.1 |
| 2016-4 | 386 | 1,200,083 | 383 (1,024) | 0.032 | 37.4 | 471 (1,024) | 0.039 | 46.0 |
| 2017-1 | 390 | 1,263,105 | 389 (927) | 0.031 | 42.0 | 466 (927) | 0.037 | 50.3 |
| 2017-2 | 400 | 1,263,981 | 308 (911) | 0.024 | 33.8 | 395 (911) | 0.031 | 43.4 |
| 2017-3 | 405 | 1,293,205 | 355 (1,157) | 0.028 | 30.7 | 424 (1,157) | 0.033 | 36.7 |
| 2017-4 | 411 | 1,293,657 | 390 (1,039) | 0.030 | 37.5 | 461 (1,039) | 0.036 | 44.4 |

Abbreviations: Carb-NS, carbapenem-nonsusceptible; MDR, multidrug resistant; Q, quarter
